# Lysophosphatidylglucoside/GPR55 signaling promotes foam cell formation in human M2c macrophages

**DOI:** 10.1038/s41598-023-39904-x

**Published:** 2023-08-06

**Authors:** Ryosuke Shimai, Kei Hanafusa, Hitoshi Nakayama, Eriko Oshima, Masaki Kato, Koki Kano, Ichiro Matsuo, Tetsuro Miyazaki, Takashi Tokano, Yoshio Hirabayashi, Kazuhisa Iwabuchi, Tohru Minamino

**Affiliations:** 1 Department of Cardiovascular Biology and Medicine, Juntendo University Graduate School of Medicine, 2-1-1, Hongo, Bunkyo-Ku, Tokyo, 113-8421 Japan; 2 Institute for Environmental and Gender-Specific Medicine, Juntendo University Graduate School of Medicine, 2-1-1, Tomioka, Urayasu, Chiba, 279-0021 Japan; 3 Infection Control Nursing, Juntendo University Graduate School of Health Care and Nursing, 2-5-1 Takasu, Urayasu, Chiba, 279-0021 Japan; 4 Laboratory for Transcriptome Technology, RIKEN Center for Integrative Medical Sciences, 2-1 Hirosawa, Wako, Saitama, 351-0198 Japan; 5 Division of Molecular Science, Gunma University, Kiryu, Gunma 376-8515 Japan; 6 RIKEN Cluster for Pioneering Research, RIKEN, 2-1, Hirosawa, Wako, Saitama 351-0198 Japan; 7 Preparation Office for Establishment of the Faculty of Pharmaceutical Science, Juntendo University, 6-8-1 Hinode , Urayasu, Chiba, 279-0013 Japan

**Keywords:** Cardiology, Cardiovascular biology, Monocytes and macrophages, Foam cells, Glycolipids, Sterols

## Abstract

Atherosclerosis is a major cause of cerebral and cardiovascular diseases. Intravascular plaques, a well-known pathological finding of atherosclerosis, have a necrotic core composed of macrophages and dead cells. Intraplaque macrophages, which are classified into various subtypes, play key roles in maintenance of normal cellular microenvironment. Excessive uptake of oxidized low-density lipoprotein causes conversion of macrophages to foam cells, and consequent progression/exacerbation of atherosclerosis. G-protein-coupled receptor 55 (GPR55) signaling has been reported to associate with atherosclerosis progression. We demonstrated recently that lysophosphatidylglucoside (lysoPtdGlc) is a specific ligand of GPR55, although in general physiological ligands of GPR55 are poorly understood. Phosphatidylglucoside is expressed on human monocytes and can be converted to lysoPtdGlc. In the present study, we examined possible involvement of lysoPtdGlc/GPR55 signaling in foam cell formation. In monocyte-derived M2c macrophages, lysoPtdGlc/GPR55 signaling inhibited translocation of ATP binding cassette subfamily A member 1 to plasma membrane, and cholesterol efflux. Such inhibitory effect was reversed by GPR55 antagonist ML193. LysoPtdGlc/GPR55 signaling in M2c macrophages was involved in excessive lipid accumulation, thereby promoting foam cell formation. Our findings suggest that lysoPtdGlc/GPR55 signaling is a potential therapeutic target for inhibition of atherosclerosis progression.

## Introduction

Atherosclerosis, a chronic inflammatory disorder involving innate and adaptive immune responses^1^, is a major cause of cerebral and cardiovascular diseases, and its incidence is steadily increasing. In 2019, ischemic heart disease and stroke were the leading and second-leading causes of death worldwide^2,3^. Intravascular plaques, a characteristic pathological finding of atherosclerosis, form a necrotic core composed of macrophages and dead cells^4,5^. In early stages of atherosclerosis development, lipoprotein permeability is enhanced by oxidized low-density lipoprotein (oxLDL)-induced inflammatory damage to arterial endothelial cells. Circulating monocytes and several types of resident tissue cells invade the vascular intima, and monocytes differentiate into various macrophage subtypes^6^. Intraplaque macrophages play key roles in maintenance of normal cellular microenvironment through their involvement in immunomodulation, lipid metabolism, and efferocytosis (clearance of apoptotic cells). However, macrophages can be converted to foam cells by excessive lipid (particularly LDL) uptake, thereby promoting atherosclerosis development. Foam cells may further trigger a cascade of lipoprotein retention, extracellular matrix modification, and inflammatory responses resulting in persistent chronic inflammation^6^. Foam cell formation is associated with decreased phagocytic activity and increased proinflammatory cytokine production, leading to progression/exacerbation of atherosclerosis^7,8^.

Intraplaque macrophages are classified into several subtypes. Activated macrophages are traditionally divided into two categories, M1 and M2 macrophages, respectively involved in pro-inflammatory and anti-inflammatory responses. There are four subtypes of M2 macrophages: M2a, M2b, M2c, and M2d. Other phenotypes relevant to atherosclerosis development have been described^7^. M1 macrophages promote atherosclerotic plaque formation based on sustained inflammation, whereas M2 macrophages cause atherosclerotic plaque regression by promoting tissue repair, anti-inflammatory cytokine release, and efferocytosis^9^. M2c macrophages, in particular, can suppress innate inflammation by migrating to affected areas, efferocytosing early apoptotic cells, and releasing anti-inflammatory cytokines^10^. However, roles of the various macrophage subtypes in atherosclerosis are not well understood.

G-protein-coupled receptor 55 (GPR55), an orphan class A G protein-coupled receptor identified in 1999^11^, recognizes multiple ligands and is associated with many physiological and pathophysiological processes that affect the central nervous system, cardiovascular system, bone remodeling, immune system control, gastrointestinal and metabolic function, and adipose tissue control^11^. In a mouse model of enteritis, GPR55-deficient mice displayed reduced levels of inflammation and macrophage infiltration^12^. GPR55 is highly expressed in human inflammatory cells (monocytes and macrophages) during atherosclerosis initiation and progression. Studies of pro-atherogenic functions of GPR55 in phorbol-12-myristate-13-acetate (PMA)-differentiated human THP-1 macrophages and endothelial cells suggested involvement of GPR55-mediated alteration of macrophage gene expression in chronic inflammation associated with lipid metabolism^13,14^.

Phosphatidylglucoside (PtdGlc), a cell surface glycophospholipid originally detected in human umbilical cord erythrocytes, is involved in erythrocyte differentiation and apoptosis^15,16^. It is also expressed on cell surfaces of phagocytes (neutrophils, monocytes) and plays a role in neutrophil differentiation and apoptosis^17,18^. Lysophosphatidylglucoside (LysoPtdGlc), a degradation product of PtdGlc, functions as a chemotactic molecule for human monocytes/macrophages via GPR55 receptor^19^. Stimulation of cells results in degradation of PtdGlc by secretory phospholipase A_2_ to produce water-soluble lysoPtdGlc^20^. Our 2022 study revealed that UDP-glucose glycoprotein glucosyltransferase 2 (UGGT2), the enzyme responsible for PtdGlc biosynthesis, plays a key role in regulation of lipid homeostasis^21^. Pro-atherogenic effects have been attributed to saturated fatty acids^22^. We proposed that UGGT2-dependent PtdGlc biosynthesis leads to elimination of excessive saturated lipids and reduction of hypoxia-induced lipid bilayer stress in the endoplasmic reticulum (ER)^21^.

Excessive circulating oxLDL is associated with atherosclerosis progression. oxLDL is taken up by intraplaque macrophages via scavenger receptors, including CD36 and lectin-like oxLDL receptor-1^23^. CD36 is highly expressed on macrophages in general, and levels are higher on M2 than on M1 macrophages^24,25^. Following uptake, oxLDL is degraded by lysosomal acidic lipase (LAL) in late endosomes. For storage of degradation products in lipid droplets, cholesterol and fatty acids are transported to the ER and re-esterified by sterol O-acyltransferase 1 (SOAT1)^8,26^. Stored esters are metabolized by neutral cholesterol ester hydrolase (NCEH1) to free cholesterol^26^, and resulting products (acyl glycerides and cholesteryl esters) accumulate in lipid droplets. In contrast, cytotoxic free cholesterol and fatty acids are loaded onto apolipoproteins and excreted extracellularly by two transporters: ATP binding cassette subfamily A member 1 (ABCA1) and ATP binding cassette subfamily G member 1 (ABCG1). In ABCA1- and ABCG1-induced cholesterol efflux, liver X receptors (LXRs) recognize intracellular cholesterol, a product of oxLDL^27,28^, and activate the peroxisome proliferator-activated receptor (PPAR) pathway, thereby promoting ABCA1 and ABCG1 biosynthesis^29^. This process is disrupted by foam cells, which incorporate excessive amounts of lipids, with consequent imbalance of lipid uptake, lipid metabolism, and cholesterol efflux.

In early atherosclerotic lesions, foam cells and dead cells are efficiently removed by M2c macrophages to maintain the intravascular environment^30,31^. In advanced atherosclerosis, excessive foam cell formation promotes apoptosis regardless of M2c macrophage activity, and failure to remove these cells accelerates plaque formation, leading to atherosclerosis progression^30,31^. The reasons why M2c macrophages do not display notable anti-inflammatory effects in advanced atherosclerosis lesions are unclear^31^. Most intraplaque cells, including macrophages and neutrophils, express PtdGlc^32^, and such PtdGlc-expressing cells release lysoPtdGlc into the surrounding environment^20^. LysoPtdGlc activates macrophages via GPR55^19^. The above observations suggest the possibility that PtdGlc/lysoPtdGlc/GPR55 signaling involves macrophage-mediated foam cell formation. We report here pharmacological evaluation in vitro of GPR55-mediated lysoPtdGlc effect on M2c macrophage foam cell formation.

## Results

### GPR55 mRNA expression level was higher for M2c than other macrophage subtypes

**Figure 1:**
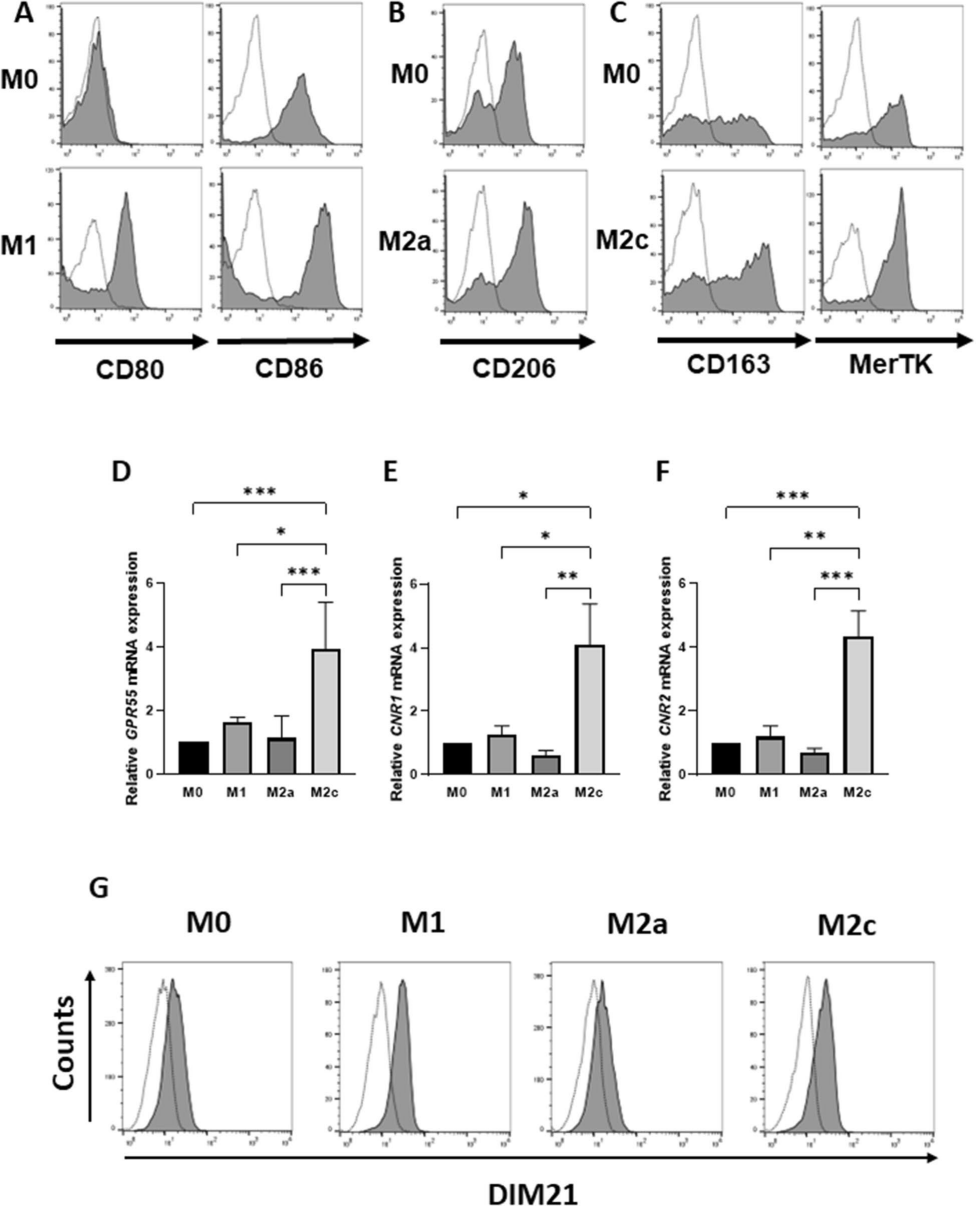
mRNA expression levels of cannabinoid receptors in various macrophage subtypes. (**A**–**C**) Human monocyte-derived macrophages (M0) were classified into three subtypes: M1 (CD80^+^CD86^+^; **A**), M2a (CD206^+^; **B**), and M2c (CD163^+^MerTK^+^; **C**). Surface expression of molecular markers was analyzed by flow cytometry, and representative histograms from three independent experiments are shown. Dotted line: isotype control. Solid line: antigen-specific antibodies. (**D**–**F**) mRNA expression of cannabinoid receptors *GPR55* (**D**), *CNR1* (**E**), and *CNR2* (**F**) was quantified by RT-qPCR. Bars represent mean ± SEM from five independent experiments. Data were analyzed by one-way ANOVA and Tukey’s multiple comparison test (*p < 0.05; **p < 0.01; ***p < 0.001; ****p < 0.0001). (**G**) M0 and polarized macrophages (M1, M2a, M2c) were stained with DIM21 antibody, which recognizes PtdGlc-enriched microdomain, followed by Alexa Fluor 488-conjugated secondary antibody. Cells were analyzed by flow cytometry, and histograms are shown. Dotted line, isotype control. Solid line, DIM21.

GPR55 agonist O-1602 was reported by V. Chiurchiu’s group to upregulate CD36 expression in THP-1 macrophages, which take up oxLDL^13^. Human monocyte-derived M2a and M2c macrophages polarized respectively by IL-4 and IL-10 displayed enhanced lipid uptake and foam cell formation^33^. Relationships in polarized human monocyte-derived macrophages between GPR55-mediated signaling and foam cell formation remain unclear. Cell surface marker protein expression under our experimental conditions was higher for polarized M1, M2a, and M2c than for M0 macrophages (Fig. 1A–C). RT-qPCR analysis revealed expression in all macrophage subtypes of cannabinoid receptors *GPR55*, type I (*CNR1*), and type II (*CNR2*). Such expression was higher for M2c than for other subtypes (Fig. 1D–F).

Human monocytes express PtdGlc^17^, and LysoPtdGlc is a specific ligand for GPR55^19^. Flow cytometric analysis using anti-PtdGlc mAb DIM21 revealed that surface PtdGlc expression was higher for M1 and M2c than for M0 or M2a macrophages (Fig. 1G).

### oxLDL uptake by macrophages induces foam cell formation

**Figure 2:**
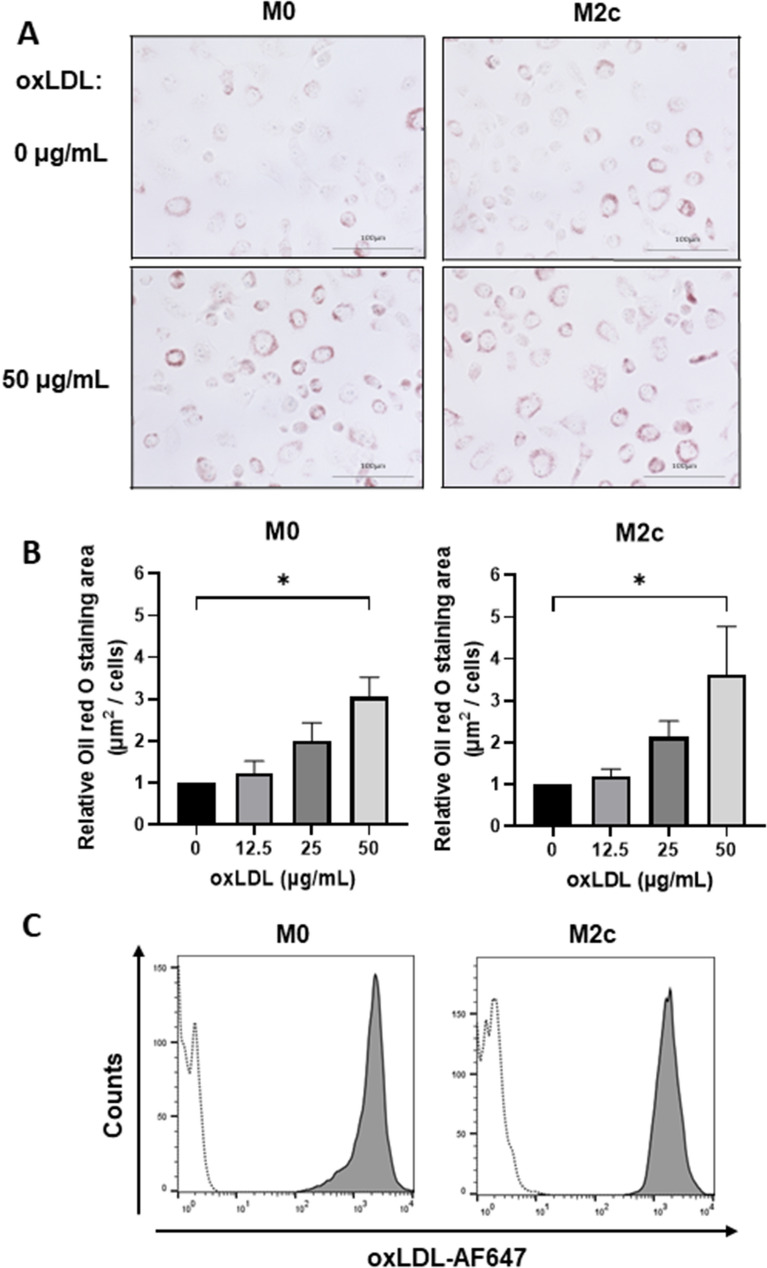
oxLDL uptake induces foam cell formation in M0 and M2c macrophages. (**A**,**B**) oxLDL uptake in macrophages was determined based on ORO staining. Cells were observed under light microscopy (40× objective lens), and ORO-stained areas were analyzed. Stained areas in M0 and M2c macrophages were normalized to ORO-stained cell number (**A**), and representative images are shown (**B**). Bars represent mean ± SEM from five independent experiments. (**C**) Alexa Fluor 647-conjugated oxLDL uptake in M0 and M2c macrophages was analyzed by flow cytometry, and representative histograms from three independent experiments are shown. Dotted and solid lines: oxLDL treatments at 0 and 50 µg/mL, respectively.

Lipid processing abilities of M0 and M2c macrophages were evaluated by staining lipid droplets from cells with ORO following oxLDL uptake (Fig. 2A, Supplement Fig. 1). ORO staining area was significantly larger in oxLDL (50 µg/mL)-treated than in nontreated cells for both M0 and M2c (Fig. 2B). Flow cytometric analysis revealed that most M0 and M2c cells took up Alexa Fluor 647-conjugated oxLDL (Fig. 2C); they were therefore defined as foam cells.

### Effects of lysoPtdGlc/GPR55 signaling on lipid uptake and efflux

**Figure 3:**
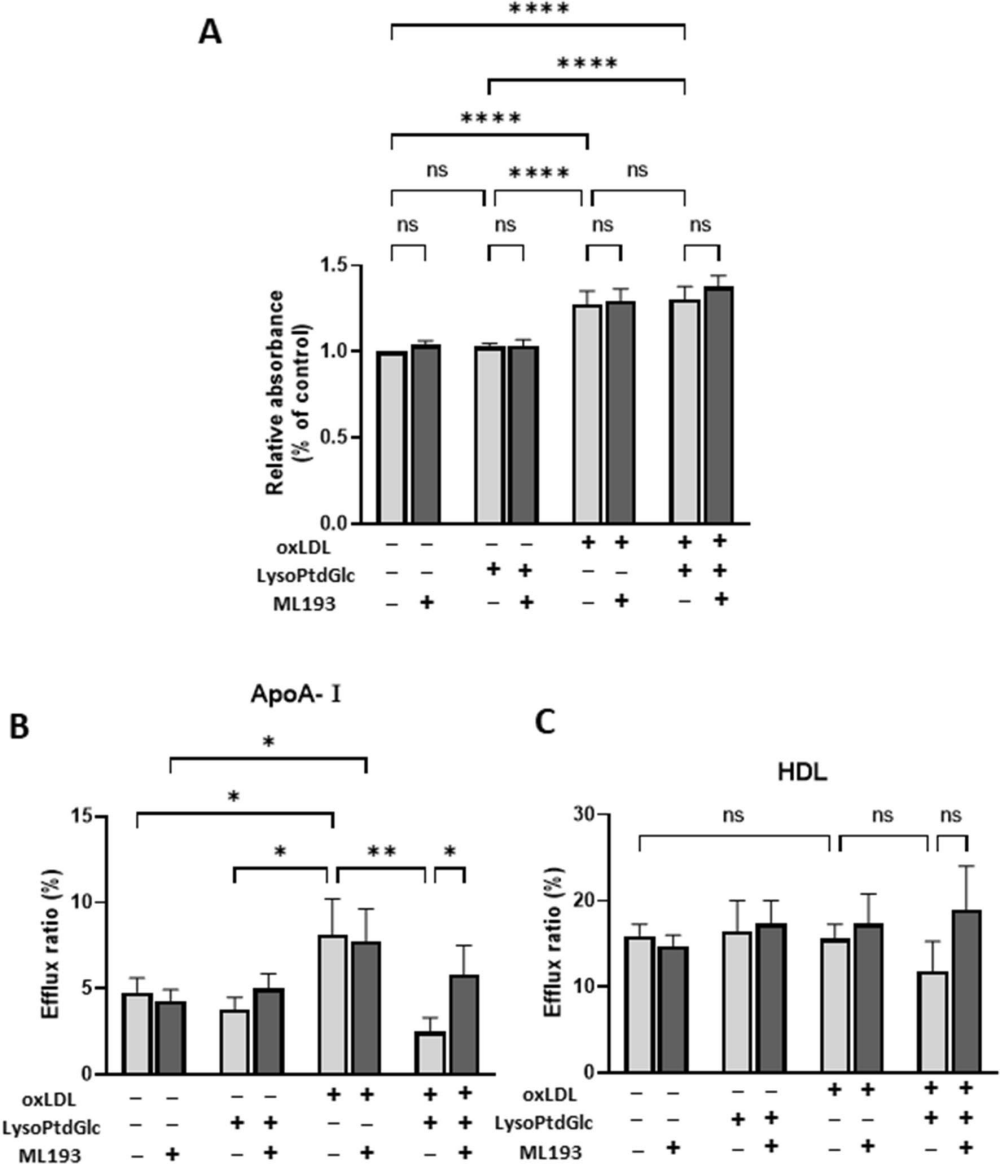
Lipid uptake and cholesterol efflux activity in lysoPtdGlc-stimulated M2c macrophages. (**A**) M2c macrophages were treated with ML193 (GPR55 antagonist) for 2 h, stimulated with 10 nM lysoPtdGlc for 2 h, incubated with 50 µg/mL oxLDL for 24 h, stained with ORO, and dissolved in isopropanol. Absorbance of supernatants was measured at wavelength 490 nm. (**B**,**C**) ABCA1-mediated (**B**) and ABCG1-mediated (**C**) cholesterol efflux in M2c macrophages was analyzed using TopFluor cholesterol under various conditions as indicated. Cells were treated sequentially with ML193, lysoPtdGlc, and oxLDL as in A. Data were normalized to non-treated cells. Bars represent mean ± SEM from three independent experiments. Data analysis and notations as in Fig. 1.

Anti-inflammatory activity of M2c macrophages in early atherosclerotic lesions is fairly efficient; in contrast, such activity in advanced atherosclerotic lesions becomes not efficient^31,34^. In view of the reported ability of LysoPtdGlc to activate macrophages^19,20^, we examined its effect on oxLDL-processing ability of M2c macrophages. Lipid accumulation was assessed by ORO staining. Intracellular lipid level in M2c macrophages was elevated by oxLDL uptake, but unaffected by lysoPtdGlc (Fig. 3A). GPR55 antagonist ML193 had no effect on oxLDL uptake. These findings indicate that lysoPtdGlc/GPR55 signaling is not involved in lipid uptake.

ABCA1 and ABCG1 are transporters that load degradation products of oxLDL, cholesterol, and phospholipids into apolipoprotein AI (ApoA-I) and high-density lipoprotein (HDL)^35^. The possible role of lysoPtdGlc/GPR55 signaling in ABCA1- or ABCG1-mediated cholesterol efflux in foamy M2c macrophages was investigated by cholesterol efflux assay (Fig. 3B,C). ABCA1-mediated cholesterol efflux was significantly elevated in these macrophages (Fig. 3B). Such efflux was significantly reduced by lysoPtdGlc stimulation of these macrophages, and the reduction was reversed by ML193 treatment. In contrast, ABCG1-mediated cholesterol efflux showed no notable elevation in foamy M2c macrophages (Fig. 3C), and was unaffected by lysoPtdGlc stimulation or ML193 treatment. These findings indicate that ABCA1-mediated (but not ABCG1-mediated) cholesterol efflux is inhibited by lysoPtdGlc/GPR55 signaling.

### Effects of lysoPtdGlc/GPR55 signaling on levels of various proteins

**Figure 4:**
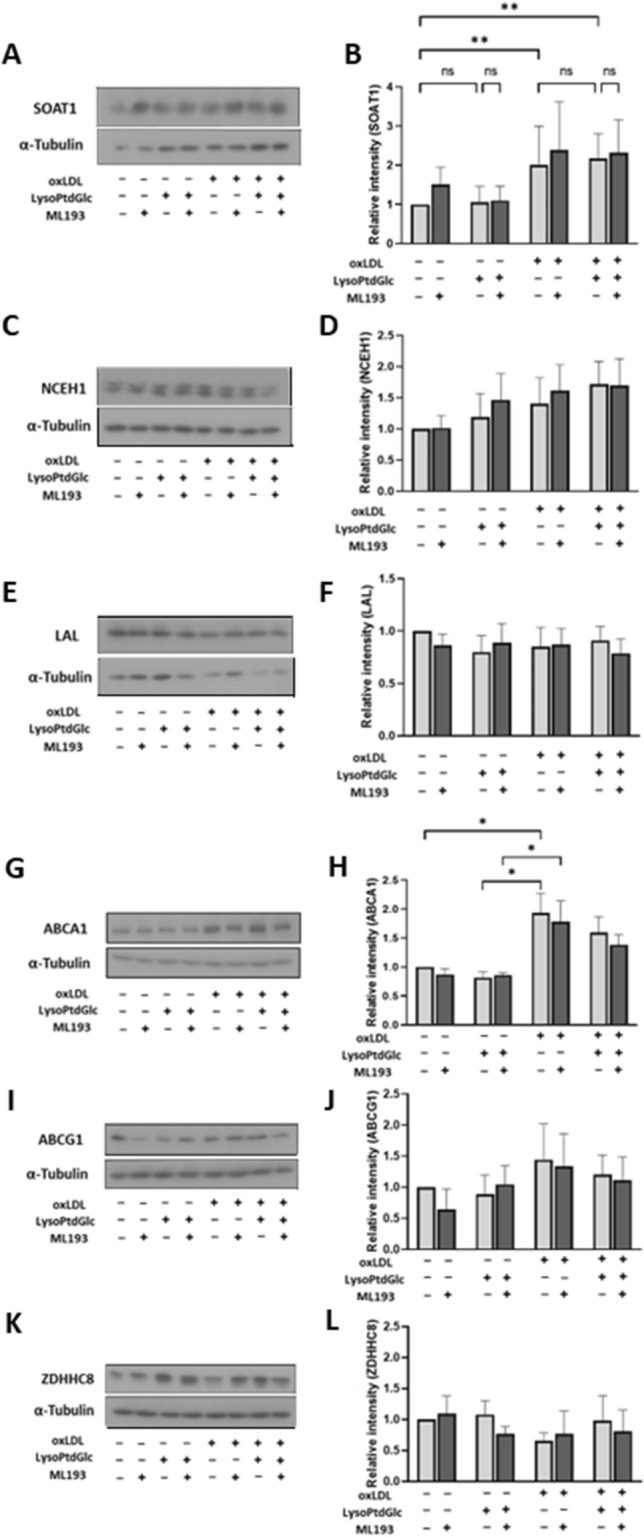
LysoPtdGlc/GPR55 signaling inhibits cholesterol efflux. (**A**–**L**) Expression of various proteins in M2c macrophages was quantified by Western blotting analysis. Representative blots of SOAT1 (**A**), NCEH1 (**C**), LAL (**E**), ABCA1 (**G**), ABCG1 (**I**), and ZDHHC8 (**K**) are shown. Relative band intensities were determined by densitometric analysis and normalized to α-tubulin (**B**, **D**, **F**, **H**, **J**, **L**). Data are presented as mean ± SEM from five independent experiments.

The ability to regulate internal lipid content affects foam cell formation of macrophages. The enzyme LAL (see “Introduction” section) hydrolyzes oxLDL in late endosomes, SOAT1 esterifies cholesterol and fatty acids in ER to store oxLDL degradation products in lipid droplets, and NCEH1 metabolizes stored esters in ER to free cholesterol. These are important processes in metabolism of lipids taken up by macrophages^23,25,36^. We examined effects of lysoPtdGlc on protein expression of these three enzymes in M2c macrophages. SOAT1 expression was significantly increased by oxLDL uptake, but was unaffected by lysoPtdGlc in the presence or absence of oxLDL uptake, and also unaffected by ML193 (Fig. 4A,B). Expressions of LAL and NCEH1 were unaffected by oxLDL, lysoPtdGlc, ML193, or combination treatments (Fig. 4C–F). These findings indicate that lysoPtdGlc/GPR55 signaling is not involved in SOAT1-, NCEH1-, or LAL-mediated lipid metabolism.

ABCA1 protein expression in M2c macrophages was significantly increased by oxLDL uptake, but was unaffected by lysoPtdGlc in the presence or absence of oxLDL uptake, and also unaffected by ML193 (Fig. 4G,H). ABCG1 expression was not significantly increased by oxLDL uptake, and was unaffected by lysoPtdGlc or ML193 (Fig. 4I,J).

ABCA1 is localized in intracellular vesicles and translocated to the cell surface through palmitoylation by Zinc finger DHHC-domain-containing protein 8 (ZDHHC8), a palmitoyltransferase^37^. We therefore examined ZDHHC8 protein expression, and found that it was unchanged by oxLDL, lysoPtdGlc, ML193, or combination treatment (Fig. 4K,L). These findings, taken together, indicate that lysoPtdGlc-mediated downregulation of ABCA1-dependent cholesterol efflux in M2c macrophages is independent of alteration of ABCA1 protein expression induced by oxLDL uptake, and that lysoPtdGlc plays a role in ABCA1- but not ABCG1-mediated cholesterol efflux.

### LysoPtdGlc/GPR55 signaling inhibits translocation of ABCA1 to plasma membranes

**Figure 5:**
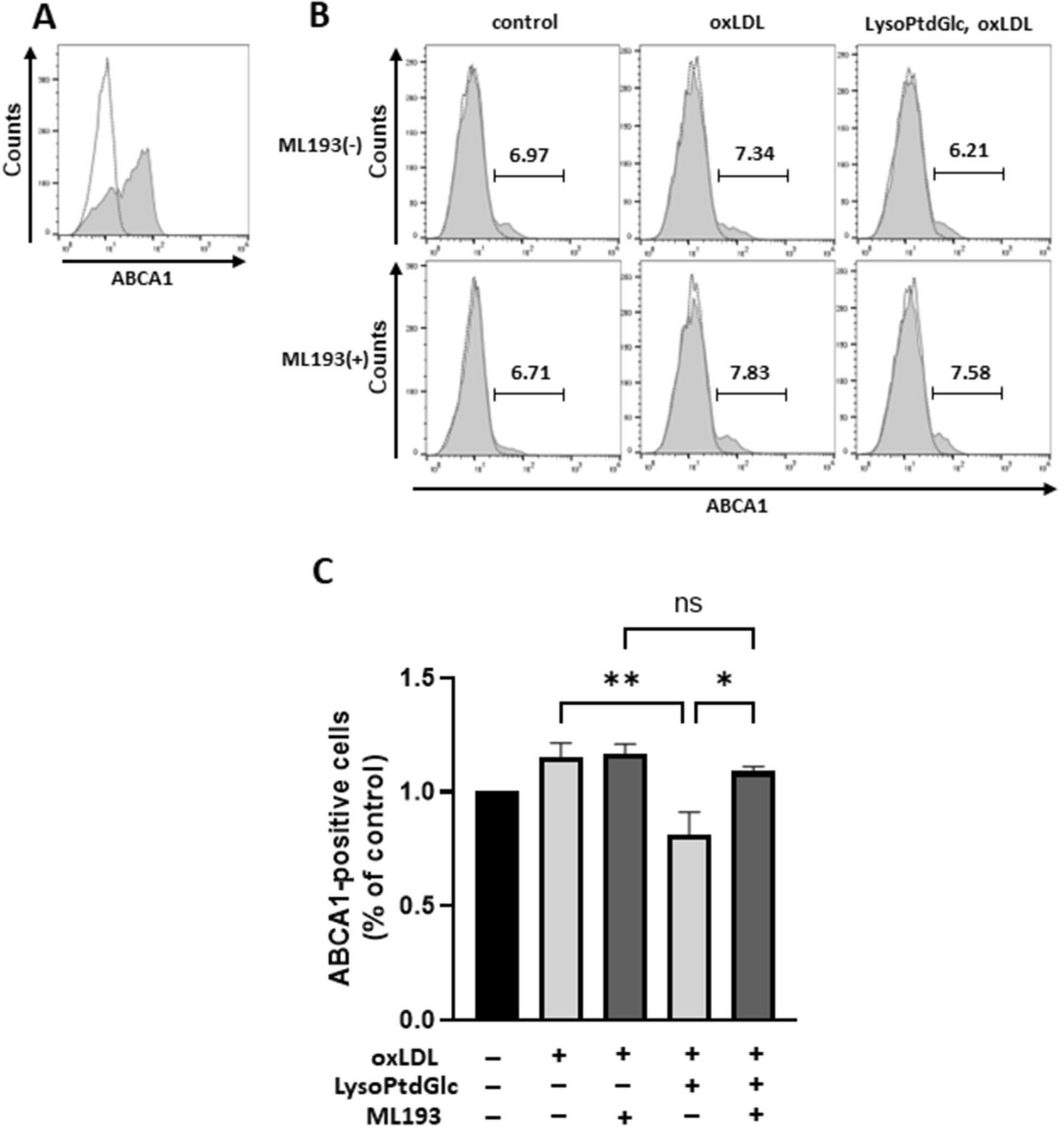
LysoPtdGlc/GPR55 signaling reduces surface expression of ABCA1 on M2c macrophages. ABCA1 expression in M2c macrophages was analyzed by flow cytometry. (**A**) Cells were fixed, permeabilized with digitonin, and stained with anti-ABCA1 antibody. And then, the cells were stained with Alexa Fluor 488 conjugated secondary antibody. A representative histogram from three independent experiments is shown. Dotted line, isotype control. Solid line, anti-ABCA1 antibody. (**B**,**C**) ABCA1 surface expression was analyzed by flow cytometry. Cells were treated with ML193 for 2 h, stimulated with 10 nM lysoPtdGlc for 2 h, and incubated with 50 µg/mL oxLDL for 24 h. Representative histograms from three independent experiments are shown (**B**). Percentages of ABCA1-positive cells were normalized to those of non-treated cells (**C**). Bars represent mean ± SEM from three independent experiments.

In view of experimental results described in “Effects of lysoPtdGlc/GPR55 signaling on levels of various proteins” section, we examined the possibility that lysoPtdGlc/GPR55 signaling suppresses ABCA1 translocation from intracellular compartments to the cell surface^38–41^, through flow cytometric analysis of surface ABCA1 expression in M2c macrophages. ABCA1 expression was high in intracellular compartments (Fig. 5A) but much lower on cell surfaces (Fig. 5B). Number of ABCA1-positive cells was increased by oxLDL uptake, and ML193 had no effect on such increase (Fig. 5C). The oxLDL-induced increase of ABCA1-positive cell number was inhibited by lysoPtdGlc, and such inhibition was reversed by ML193. Surface expression of ABCG1 on M2c macrophages was not upregulated by oxLDL uptake, nor by lysoPtdGlc stimulation (Supplement Fig. 2). These findings indicate that lysoPtdGlc/GPR55 signaling suppresses ABCA1 translocation to the cell surface, and thereby inhibits ABCA1-mediated cholesterol efflux in foamy M2c macrophages.

## Discussion

In macrophages, oxLDL is taken up, internalized, metabolized, and exported as cholesterol. Based on cholesterol efflux assay, we demonstrated the essential role of lysoPtdGlc/GPR55 signaling in cholesterol efflux in M2c macrophages. lysoPtdGlc did not affect cholesterol uptake in these cells. LysoPtdGlc/GPR55 signaling had no effect on expression of LAL, SOAT1, or NCEH1 proteins, which are involved in metabolism of oxLDL. Present and previous findings^13^, considered together, suggest that cholesterol efflux in atherosclerosis-associated M2c macrophages is downregulated by lysoPtdGlc stimulation. Pretreatment with lysoPtdGlc inhibits ABCA1-mediated cholesterol efflux in foamy M2c macrophages, which take up excessive amounts of oxLDL. ABCA1 surface expression is inhibited by lysoPtdGlc/GPR55 signaling, although such signaling is not directly involved in biosynthesis of ABCA1 protein. Cholesterol induces PPARγ-LXRα pathway and upregulates ABCA1 and ABCG1 expression^35^. Cholesterol efflux is promoted by cAMP/PKA-induced phosphorylation at the cell surface^42^. Present findings indicate that lysoPtdGlc/GPR55 signaling is associated with translocation of ABCA1 to the cell surface, but not with PPARγ-LXRα or cAMP/PKA pathway. ABCA1 translocation is regulated by palmitoyltransferase ZDHHC8^37^; however, lysoPtdGlc/GPR55 signaling had no effect on ZDHHC8 protein level. Mechanisms underlying regulation of ABCA1 translocation by lysoPtdGlc/GPR55 signaling via ZDHHC8 remain to be elucidated.

Cell surface PtdGlc expression on several macrophage subtypes was demonstrated by our experiments. PtdGlc is expressed by most intraplaque cells^32^. Activated macrophages, vascular endothelial cells, and vascular smooth muscle cells all induce apoptosis in plaque^43^. Neutrophils, which induce apoptosis via PtdGlc^17^, are localized at plaque erosion sites^44^. It is therefore conceivable that lysoPtdGlc is released from intraplaque cells during their activation and apoptosis. V. Chiurchiu’s group reported increased GPR55 expression in THP-1 macrophages during foam cell formation^13^. GPR55 agonist O-1602 increased oxLDL-induced lipid accumulation in these cells by upregulating CD36 and scavenger receptor Class B Type I, but decreased cholesterol efflux by downregulating ABCA1 and ABCG1^13^. Adhesion of THP-1 monocytes to vascular endothelial cells was enhanced by O-1602, with consequent promotion of atherosclerosis^14^, upregulation of proinflammatory TNF-α protein, and downregulation of anti-inflammatory IL-10^13^. On the other hand, Y. Yin’s group reported that GPR55 antagonist CID16020046 in human aortic endothelial cells inhibited oxLDL-induced apoptosis, secretion of inflammatory cytokines IL-8 and monocyte chemoattractant protein-1 (MCP-1), and oxLDL-induced expression of the adhesion molecules vascular cell adhesion molecule-1 (VCAM-1) and E-selectin^45^. These pharmacological results are consistent with a pro-atherosclerotic role of GPR55, with our observation of promotion of atherosclerosis progression by GPR55, and with the proposed involvement of lysoPtdGlc/GPR55 signaling in macrophages and foam cells in lipid accumulation and atherosclerosis progression.

Palmitoylethanolamide (PEA), an endogenous fatty acid amide, is another ligand for GPR55, and promotes efferocytosis of M2c macrophages and reduction of plaque size in early atherosclerotic lesions^46^. In contrast, in our proposed advanced atherosclerosis model, foam cell formation is promoted by lysoPtdGlc/GPR55 signaling. Excessive lipid uptake suppresses efferocytosis of M2c macrophages, leading to their conversion to foam cells^34^. GPR55-mediated functions of M2c macrophages may thus change from PEA-dependent anti-inflammatory effect to lysoPtdGlc-dependent promoting effect on atherosclerosis development. To elucidate the involvement of the GPR55 signal in the regulation of lipid metabolism homeostasis and promotion of atherosclerosis progression in human atherosclerotic lesions, it is necessary to determine the levels of lysoPtdGlc and the expression variability of GPR55 in atherosclerotic tissue. However, lysoPtdGlc is thought to be present only in trace amounts in biological samples^20^. Since lysophospholipids are readily degraded by phospholipases, it is difficult to obtain sufficient amounts of lysoPtdGlc from human atherosclerotic lesions without degradation. In addition, current analytical techniques have not established a method for quantitatively analyzing lysoPtdGlc at trace levels in biological samples under the coexistence of other lysophospholipids. In the future, it is necessary to establish analytical techniques that enable quantitative analysis of lysoPtdGlc in atherosclerotic lesions. In addition, to clarify the molecular mechanism of how lysoPtdGlc and GPR55 are involved in the progression of atherosclerosis, further detailed analysis is needed in an atherosclerosis model using GPR55-deficient mice.

In this study, we demonstrated the GPR55-mediated effects of lysoPtdGlc on M2c macrophage foam cell formation in vitro. The results show that lysoPtdGlc/GPR55 signaling inhibits ABCA1-mediated cholesterol efflux in foamy M2c macrophages via inhibition of ABCA1 surface expression. These results suggest that lysoPtdGlc/GPR55 signaling is a potential therapeutic target for inhibiting atherosclerosis progression. Further studies on the molecular mechanisms involving lysoPtdGlc/GPR55 signaling in M2c macrophage-mediated atherosclerosis progression are warranted.

## Materials and methods

### Reagents

LysoPtdGlc was synthesized according to previous reports^20,47,48^ and briefly described in Supplementary Materials and Methods. OxLDL from human plasma was from Thermo Fisher Scientific (Waltham, Germany). Lipopolysaccharide (LPS) derived from *Escherichia coli* O111:B4 was from Sigma-Aldrich (St. Louis, MO, USA). Recombinant human macrophage colony-stimulating factor (M-CSF), interferon-gamma (IFN-γ), and interleukin (IL)-4 were from R&D Systems (Minneapolis, MN, USA). Phycoerythrin (PE)-conjugated anti-CD80, anti-CD86, anti-CD163, anti-CD206, and anti-MerTK monoclonal antibodies (mAbs) were from BioLegend (San Diego, CA, USA). Rabbit anti-human ABCA1 mAb, rabbit anti-human MerTK mAb, and rabbit anti-human LAL polyclonal antibody (pAb) were from Abcam (Cambridge, UK). Rabbit anti-human ABCG1 pAb was from GeneTex (Taipei, Taiwan). Rabbit anti-human ZDHHC8 and rabbit anti-human arylacetamide deacetylase-like 1 (AADACL1) pAbs were from Proteintech (Chicago, IL, USA). Rabbit anti-human SOAT1 pAb was from Cell Signaling Technology (Danvers, MA, USA). HRP-conjugated goat anti-rabbit and anti-mouse pAbs were from Chemicon (Merck Millipore; Burlington, MA, USA). Anti-PtdGlc mouse monoclonal antibody DIM21 was prepared as described previously^49^.

### Cell culture

Ethical approval for obtaining blood from healthy human volunteers was provided by the Ethics Review Board of Juntendo University Faculty of Medicine (Authorization number: 2017170). All research was performed in accordance with the Declaration of Helsinki, and relevant guidelines/regulations.

Peripheral blood was obtained from healthy volunteer subjects, with written informed consent. Peripheral blood mononuclear cells (PBMCs) were isolated from blood samples using Lymphoprep (Stemcell Technologies; Cologne, Germany) as per manufacturer’s protocol. PBMCs were suspended in DMEM/F-12 (Life Technologies; Carlsbad, CA, USA), plated on 6-well tissue culture plates (density 2 × 10^6^ cells/mL), and incubated 3 h at 37 °C. Adherent cells were cultured in RPMI 1640 supplemented with 10% FBS (Biowest; Hiroshima, Japan) and 20 ng/mL M-CSF in 6-well plates for 6 days to induce differentiation into M0 macrophages. M1 macrophages were produced by culturing M0 macrophages for 24 h in RPMI 1640/10% FBS in the presence of 1 ng/mL LPS and 20 ng/mL IFN-γ. M2a and M2c macrophages were produced by culturing M0 macrophages for 24 h in RPMI 1640/10% FBS in the presence of (respectively) 20 ng/mL IL-4 and 100 nM dexamethasone.

### Foam cells

M0 and M2c macrophages were incubated with oxLDL at various concentrations for 24 h and stained with Oil Red O solution (ORO) (ScyTek Laboratories; Logan, UT, USA) as per manufacturer’s protocol. Cells were observed using a fluorescence microscope (model BZ-X800; Keyence; Osaka, Japan) equipped with 40× objective lens, and lipid droplet area was calculated using software program BZ-H4C (Keyence). Data were expressed as ratio of lipid droplet area to cell number in 10 randomly selected fields. Lipid quantity was determined using a microplate reader (2030 ARVO X4; PerkinElmer Japan; Tokyo) to measure absorbance at wavelength 490 nm of isopropanol-extracted supernatants from ORO-stained cells.

### LysoPtdGlc stimulation and oxLDL uptake

To evaluate effects of lysoPtdGlc on functions of the four macrophage subtypes under cholesterol homeostasis in advanced atherosclerosis, polarized macrophages were pretreated for 2 h with 1 μM ML193, stimulated for 2 h with 10 nM lysoPtdGlc, incubated for 24 h with 50 μg/mL oxLDL, collected, and used for subsequent experiments.

Cholesterol efflux activity of M2c macrophages was analyzed by fluorescent TopFluor cholesterol (Avanti Polar Lipids; Alabaster, AL, USA) assay as described by H.L. Wilson’s group^33^. Macrophages (2.5 × 10^4^ cells/well) were plated on 96-well black plates, pretreated with lysoPtdGlc, and incubated sequentially with oxLDL for 24 h, 2.5 μM TopFluor cholesterol in serum-free RPMI 1640 for 2 h, and either 50 µg/mL human high-density lipoprotein (Sigma-Aldrich) or 20 µg/mL apolipoprotein AI (Sigma-Aldrich). TopFluor cholesterol fluorescence in supernatants and cells was measured with the microplate reader (excitation wavelength 490 nm; emission wavelength 520 nm).

### Quantitative reverse transcription PCR (RT-qPCR)

Cells were washed 3 × with PBS, suspended in TRIzol reagent (Life Technologies; Waltham, MA, USA), total RNA was extracted as per manufacturer’s protocol, and RT-qPCR was performed as described previously^50^. cDNA was generated using PrimeScript RT reagent kit (Takara Bio; Shiga, Japan), and RT-qPCR was performed with ABI 7900HT sequence detection system (Life Technologies) using TB Green Extaq II and specific primers (Takara Bio) (Supplement Table 1). Data were analyzed using software program SDS V. 2.4 (Life Technologies).

### Flow cytometric analysis

Polarized macrophages were detached with Accutase (Innovative Cell Technologies; San Diego, CA, USA), pretreated with FcR blocker (Miltenyi Biotec; Bergisch Gladbach, Germany), stained for 30 min at 4 °C with PE-conjugated anti-CD80, anti-CD86, anti-CD206, anti-CD163, anti-MerTK, anti-ABCA1, or anti-ABCG1 antibody (or respective isotype control antibody), washed 2 × with PBS, and subjected to flow cytometric analysis (FACSCalibur, BD Biosciences; Franklin Lakes, NJ, USA). Data were analyzed using software program FlowJo V. 10 (TreeStar; Woodburn, OR, USA).

### Western blotting analysis

Samples were run on 10% SDS-PAGE and transferred to PVDF membrane. Membranes were incubated with anti-ABCA1, anti-ABCG1, anti-ZDHHC8 (ratio 1:500), anti-human LAL, anti-human SOAT1, anti-AADACL1 (ratio 1:1000), or anti-α-tubulin (ratio 1:10,000) antibodies overnight at 4 °C, washed with TBS-T (10 mM Tris–HCl (pH 8.0) and 150 mM NaCl containing 0.05% Tween 20), incubated with HRP-conjugated secondary antibody (ratio 1:4000), and stripped by stripping buffer (62.5 mM Tris–HCl (pH 6.8), 100 mM β-mercaptoethanol, 2% SDS) for 30 min at 55 °C. X-ray film was exposed to chemical luminescence (SuperSignal reagent; Thermo Fisher) and scanned. Chemiluminescence signal intensities were quantified using software program ImageJ V. 2.1.0.

### Statistical analysis

Data were expressed as mean ± SEM and analyzed by one-way analysis of variance (ANOVA), followed by Tukey’s multiple comparison test, using software program with GraphPad (San Diego, CA) Prism software program V. 9 (San Diego, CA). The criterion for statistical significance of differences between means was *p* < 0.05.

### Supplementary Information

Supplementary Information.

## Data Availability

The datasets generated during and/or analyzed during the current study are available from the corresponding author on reasonable request.
