# Supplementary material for: Lysophosphatidylglucoside/GPR55 signaling promotes foam cell formation in human M2c macrophages
Source: Sci Rep. 2023 Aug 6;13:12740. doi: 10.1038/s41598-023-39904-x (PMC10404585; doi:10.1038/s41598-023-39904-x)
Supplement: Supplementary file 1 — Supplementary Information. [file 41598_2023_39904_MOESM1_ESM.pdf]

Supplementary Information for "Lysophosphatidylglucoside/GPR55 signaling promotes foam cell formation in human M2c macrophages"

Ryosuke Shimai[1, 2], Kei Hanafusa[2], Hitoshi Nakayama[2, 3, 4], Eriko Oshima[2], Masaki Kato[5], Koki Kano[6], Ichiro Matsuo[6], Tetsuro Miyazaki[1], Takashi Tokano[1], Yoshio Hirabayashi[7], Kazuhisa Iwabuchi[2, 3, 4, *], Tohru Minamino[1]

[1] Department of Cardiovascular Biology and Medicine, Juntendo University Graduate School of Medicine, 2-1-1, Hongo, Bunkyo-Ku, Tokyo 113-8421, Japan

[2] Institute for Environmental and Gender-specific Medicine, Juntendo University Graduate School of Medicine, 2-1-1, Tomioka, Urayasu, Chiba 279-0021, Japan

[3] Laboratory of Biochemistry, Juntendo University Faculty of Health and Nursing, 2-5-1 Takasu, Urayasu, Chiba 279-0021, Japan

[4] Infection Control Nursing, Juntendo University Graduate School of Health and Nursing, 2-5-1 Takasu, Urayasu, Chiba 279-0021, Japan

[5] Laboratory for Transcriptome Technology, RIKEN Center for Integrative Medical Sciences, 2-1 Hirosawa, Wako, Saitama 351-0198, Japan

[6] Division of Molecular Science, Gunma University, Kiryu, Gunma 376-8515, Japan

[7] RIKEN Cluster for Pioneering Research, RIKEN, Wako, Saitama 351-0198, Japan.

Supplementary Materials and Methods

Structures of phosphatidylglucoside (PtdGlc) and LysoPtdGlc

PtdGlc is composed exclusively of saturated fatty acid chains with C18:0 and C20:0 at the *sn*-1 and *sn*-2 positions of the glycerol backbone [1], respectively. PtdGlc is mainly biosynthesized by Uridine diphosphate glucose: glycoprotein glucosyltransferase 2 (UGGT2)[2], and forms membrane microdomains on plasma membranes [3,4]. Stimulation of cells results in digestion of PtdGlc by secretory phospholipase A_2 to produce a water-soluble lyso-form (lysoPtdGlc) [5,6].

PtdGlc

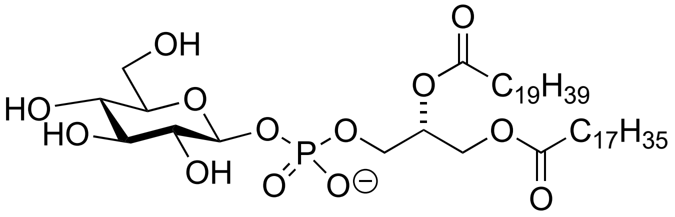

LysoPtdGlc

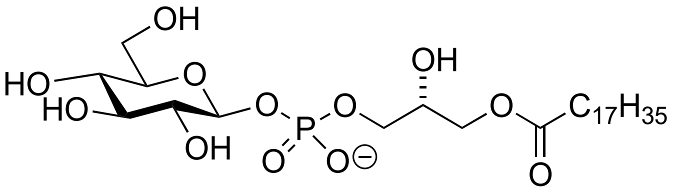

Synthesis of Lyso-PtdGlc

LysoPtdGlc was synthesized according to previous reports[6-8]. In brief, the glucosyl *H*-phosphonate moiety was synthesized via a five-step sequence from D-glucose. First, D-glucose was acetylated and then converted to the orthoester derivative **S1**. The acetyl-protecting groups of the orthoester derivative **S1** were replaced with a benzyl group. Acid-catalyzed ortho ester opening with phosphonic acid of the benzyl derivative **S2** using a microfluidic method introduced the *H*-phosphonate moiety to give the desired glucosyl *H*-phosphonate derivative **S3**. Condensation of the glycerol derivative **S4** with the prepared *H*-phosphonate derivative **S3** was achieved in a one-pot procedure by sequential pivaloyl chloride-mediated *H*-phosphonate activation and iodine oxidation to the corresponding phosphate diester derivative **S5**. Acetyl group on the glucose moiety **S5** was remover by hydrazine hydrate to give **S6**, followed by DDQ oxidation of 2-Naphthylmetyl (NAP) group on the glycerol moiety of **S6**, yielding the corresponding diol derivative **S7**. After the selective introduction of a stearoyl residue at the only

primary hydroxy group, the remaining benzyl protecting groups were removed under reducing conditions, obtaining the desired lysoPtdGlc as a white waxy solid.

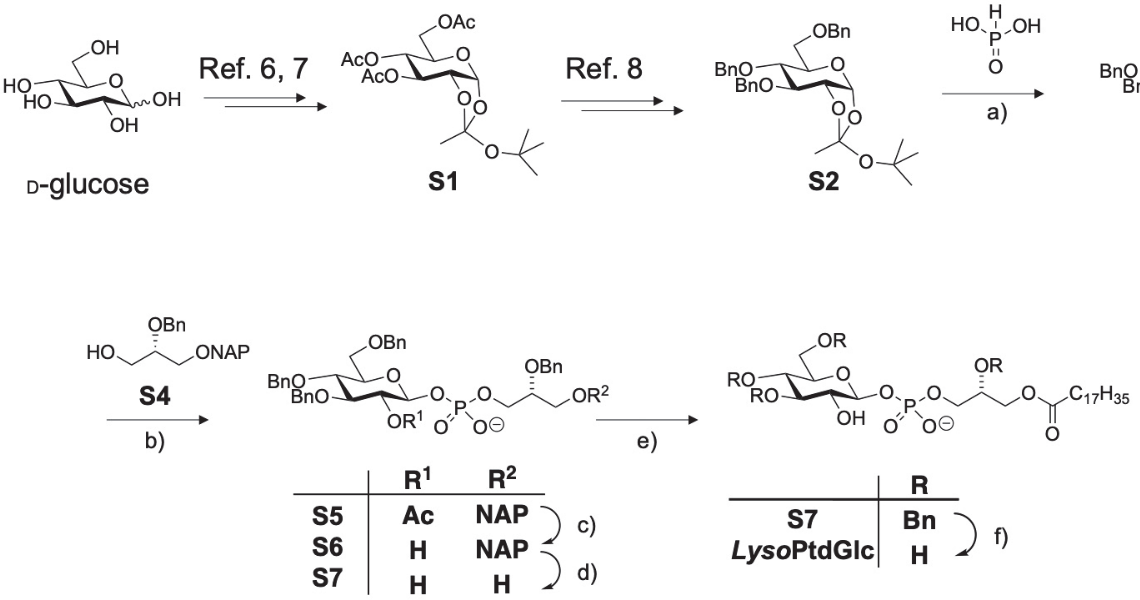

Scheme S1. Synthesis of lysoPtdGlc. Reagents and conditions: a) 1) THF, 40°C, 2) AcOH/TEA buffer pH 7, 0°C, 29% in two steps; b) 1) PivCl, pyridine, THF, 0 to 10°C, 2) 0.2 M I_2 solution, 10°C to 25 °C, 37% in two steps, c) H_2NNH_2, H_2O, MeOH, 40°C, 98 %, d) DDQ, CH_2Cl_2/H_2O=9/1, 25 °C, 79 %, e) stearic acid, EDC, DMAP, CH_2Cl_2, 25 °C, 84 %, e) H_2, Pd/C,1 eq. AcOH, EtOH, 35°C, 63%.

References:

1 Nagatsuka, Y., Tojo, H. & Hirabayashi, Y. Identification and analysis of novel glycolipids in vertebrate brains by HPLC/mass spectrometry. *Methods Enzymol.* **417**, 155-167 (2006). https://doi.org:10.1016/S0076-6879(06)17012-3

2 Hung, H. H. *et al.* Selective involvement of UGGT variant: UGGT2 in protecting mouse embryonic fibroblasts from saturated lipid-induced ER stress. *Proc. Natl. Acad. Sci. U. S. A.* **119**, e2214957119 (2022). https://doi.org:10.1073/pnas.2214957119

3 Nagatsuka, Y. *et al.* Carbohydrate-dependent signaling from the phosphatidylglucoside-based microdomain induces granulocytic differentiation of HL60 cells. *Proc. Natl. Acad. Sci. U. S. A.* **100**, 7454-7459 (2003). https://doi.org:10.1073/pnas.1232503100

4 Kinoshita, M. O. *et al.* Lipid rafts enriched in phosphatidylglucoside direct astroglial differentiation by regulating tyrosine kinase activity of epidermal growth factor receptors. *Biochem J* **419**, 565-575 (2009). https://doi.org:10.1042/BJ20081896

5 Nagatsuka, Y. *et al.* Phosphatidylglucoside exists as a single molecular species with saturated fatty acyl chains in developing astroglial membranes. *Biochemistry* **45**, 8742-8750 (2006). https://doi.org:10.1021/bi0606546

6 Guy, A. T. *et al.* NEURONAL DEVELOPMENT. Glycerophospholipid regulation of modality-specific sensory axon guidance in the spinal cord. *Science* **349**, 974-977 (2015). https://doi.org:10.1126/science.aab3516

7 Greimel, P. & Ito, Y. First synthesis of natural phosphatidyl-β-D-glucoside. *Tetrahedron Lett.* **49**, 3562–3566 (2008). https://doi.org:10.1016/j.tetlet.2008.04.036

8 Kano, K. *et al.* Stereocontrolled Synthesis of Lyso-phosphatidyl β-D-Glucoside. *ChemistrySelect* **6**, 6811–6815 (2021). https://doi.org:10.1002/slct.202102176

Supplement Table 1. Primers used for RT-qPCR.

Gene	Forward primer (5'→3')	Reverse primer (5'→3')
actb	TGGCACCCAGCACAATGAA	CTAAGTCATAGTCCGCCTAGAAGCA
GPR55	CCAGGATGCAGGTGAGTAAGA	CTGCCTTGGTTCCACCATA
CNR1	CGTTGCGGCTATCTTTGCG	CCTTCCTACCACTTCATCGGC
CNR2	GGACCCACATGATGCCCAG	GACCGCCATTGACCGATACC

Supplement Fig 1

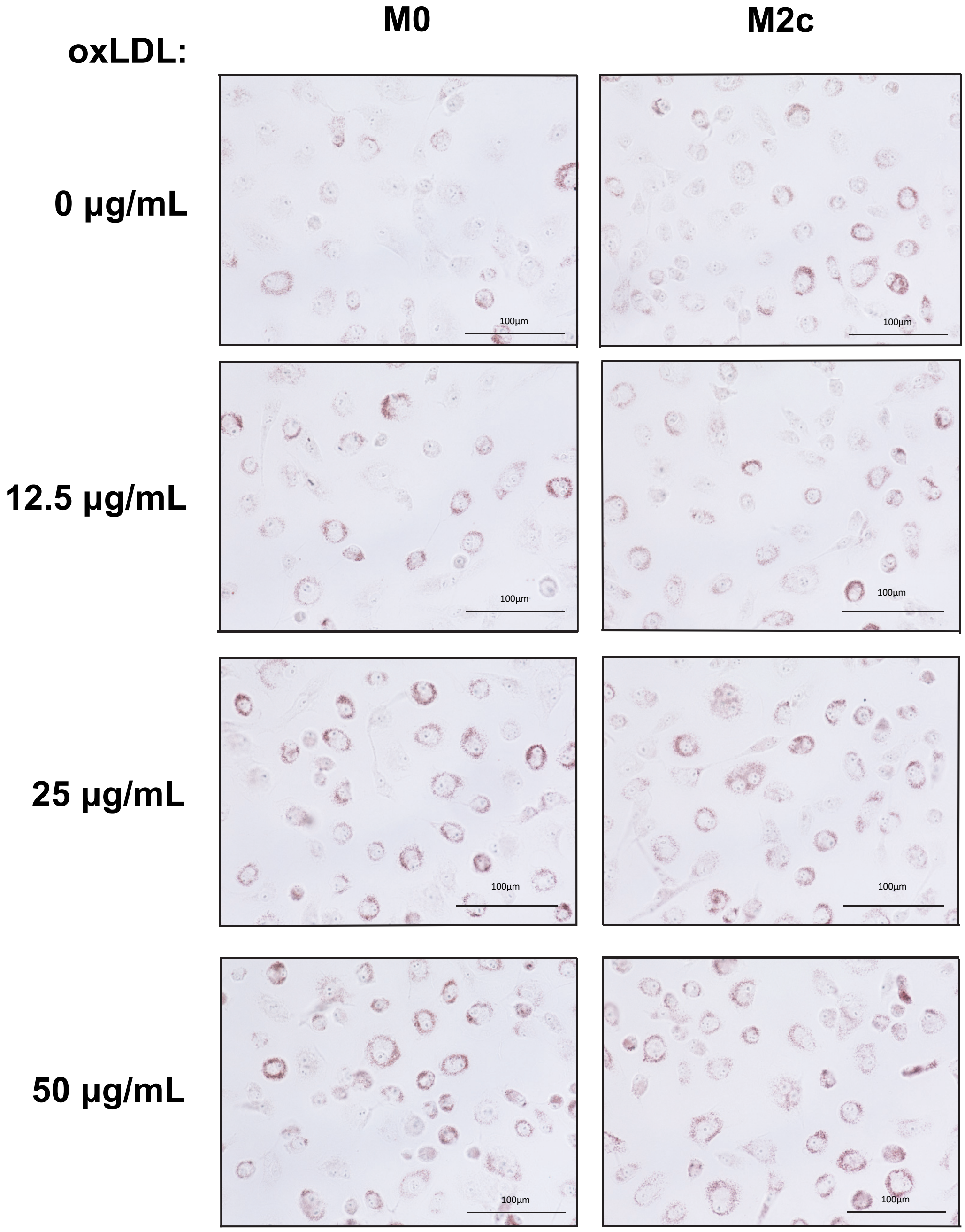

<u>Supplement Fig 1.</u> <u>Microscopic images of oxLDL-incorporated M0 and M2c macrophages</u>
M0 and M2c macrophages were incubated with the indicated concentrations of oxLDL, and stained with ORO. Stained cells were observed by light microscopy (40x objective lens). Representative images are shown.

Supplement Fig 2

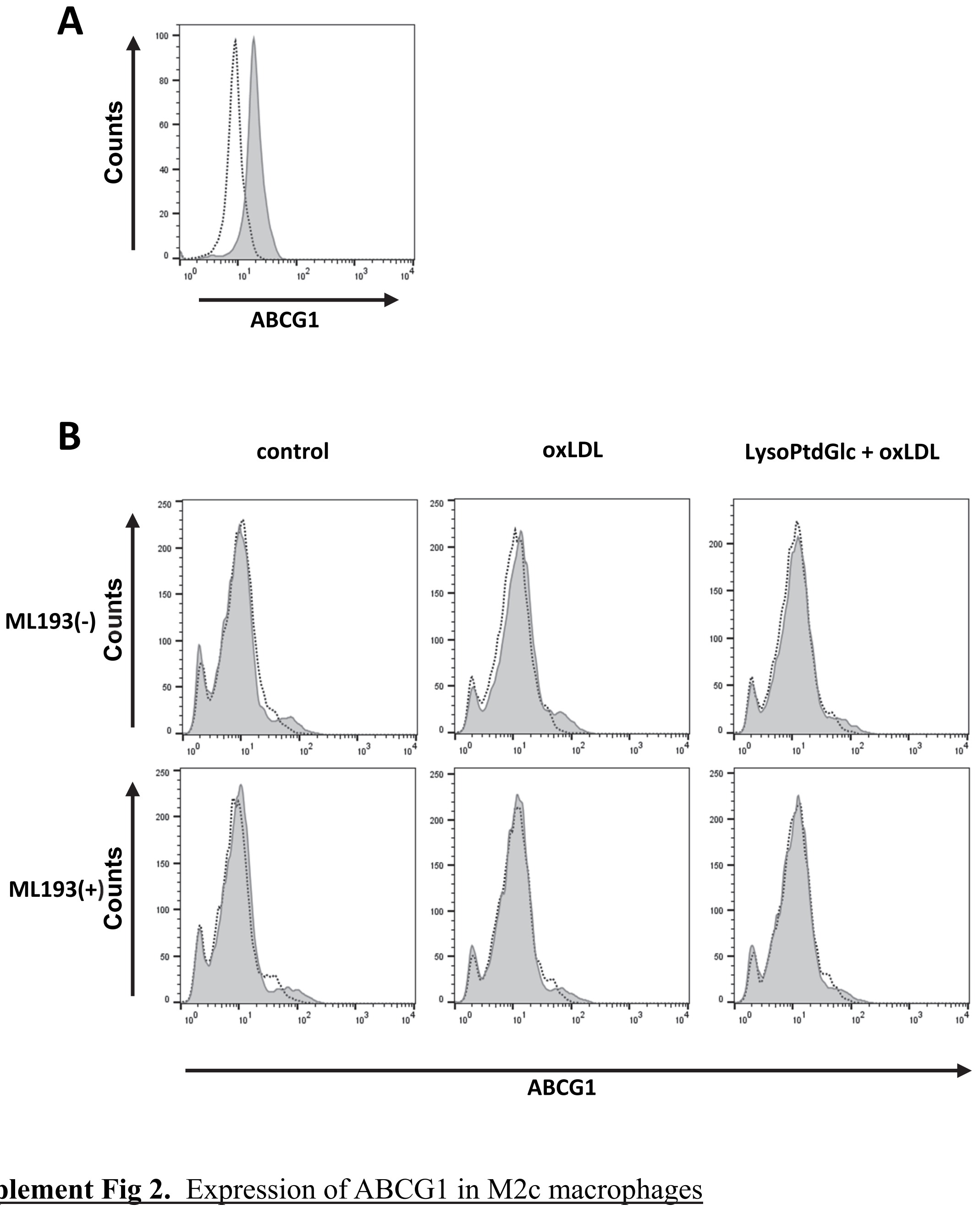

Supplement Fig 2. Expression of ABCG1 in M2c macrophages
(A) ABCG1 expression in M2c macrophages was analyzed by flow cytometry. Cells were permeabilized with digitonin, fixed, and stained with anti-ABCG1 antibody. A representative histogram from three independent experiments is shown. Dotted line, isotype control. Solid line, anti-ABCG1 antibody. (B) Surface expression of ABCG1 on M2c macrophages was analyzed by flow cytometry. Cells were treated with ML193 for 2 h, stimulated with 10 nM lysoPtdGlc for 2 h, and incubated with 50 μg/mL oxLDL for 24 h. Representative histograms from three independent experiments are shown (B).

Supplement Fig 3-1

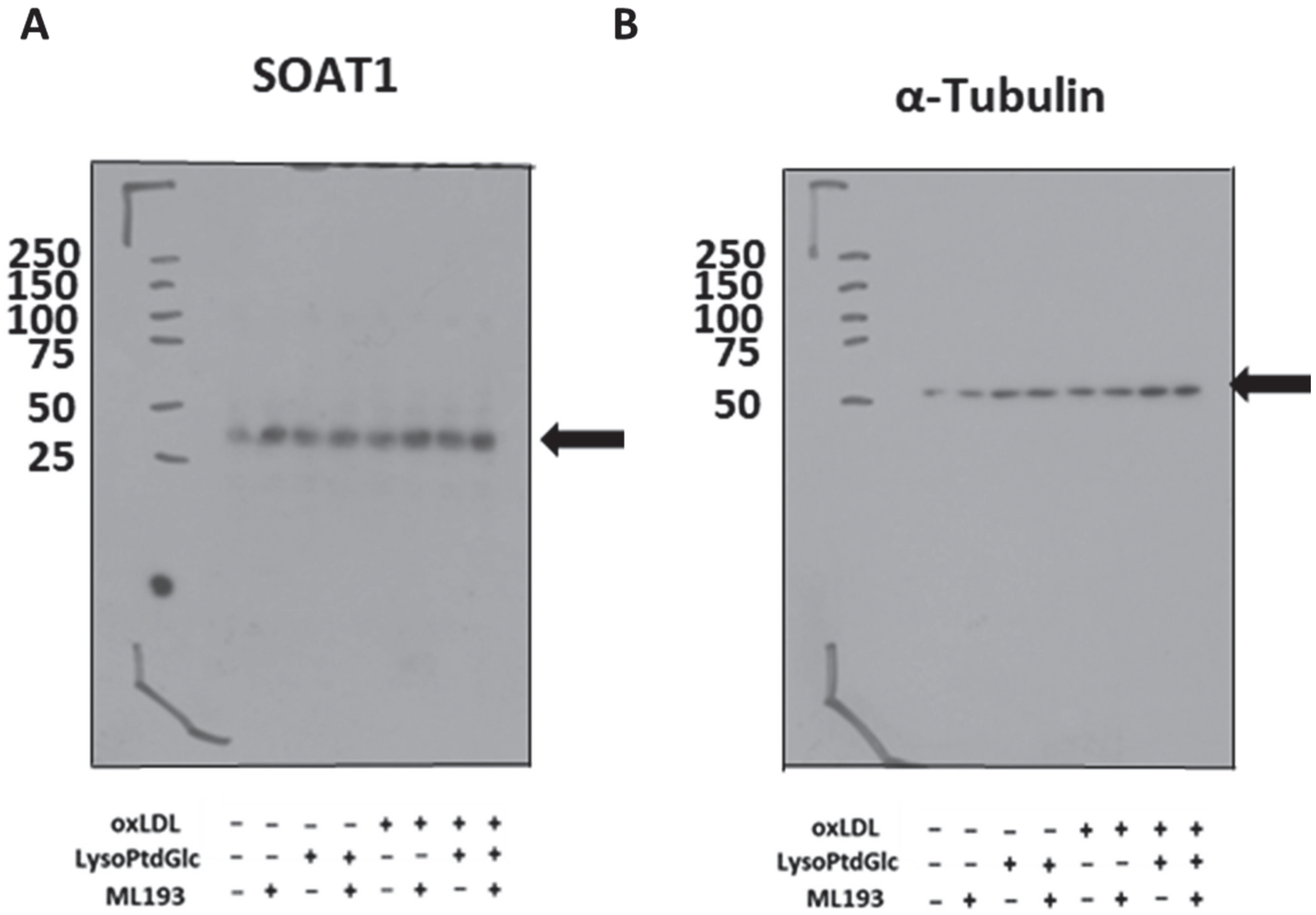

Supplement Fig 3-1. Original western blots, including protein ladders, for data presented in Fig. 4
SDS-PAGE was performed, proteins transferred onto PVDF membranes by semi-dry process, and membranes probed with appropriate HRP-conjugated primary and secondary antibodies (see M&M). Molecular weights (kDa) are indicated at left.
A, SOAT1 B, α-tubulin.

Supplement Fig 3-2

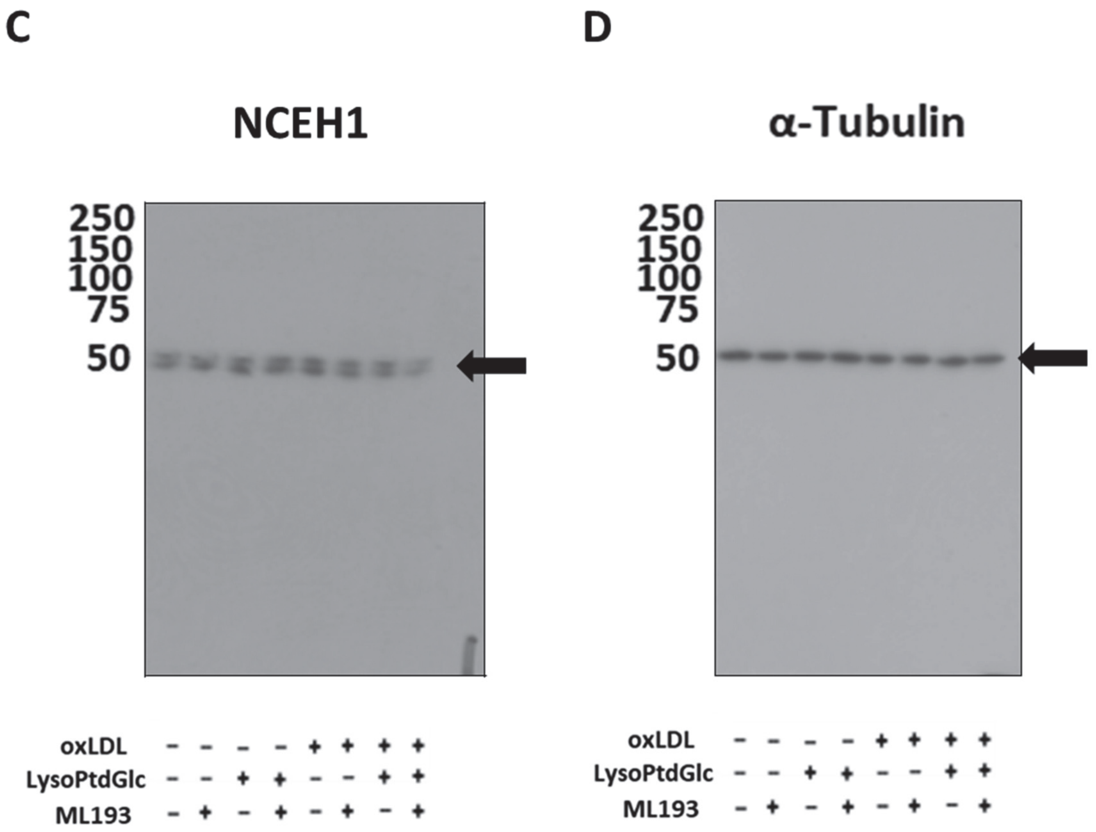

Supplement Fig 3-2. Original western blots, including protein ladders, for data presented in Fig. 4
SDS-PAGE was performed, proteins transferred onto PVDF membranes by semi-dry process, and membranes probed with appropriate HRP-conjugated primary and secondary antibodies (see M&M). Molecular weights (kDa) are indicated at left.
C, NCEH1 D, α-tubulin.

Supplement Fig 3-3

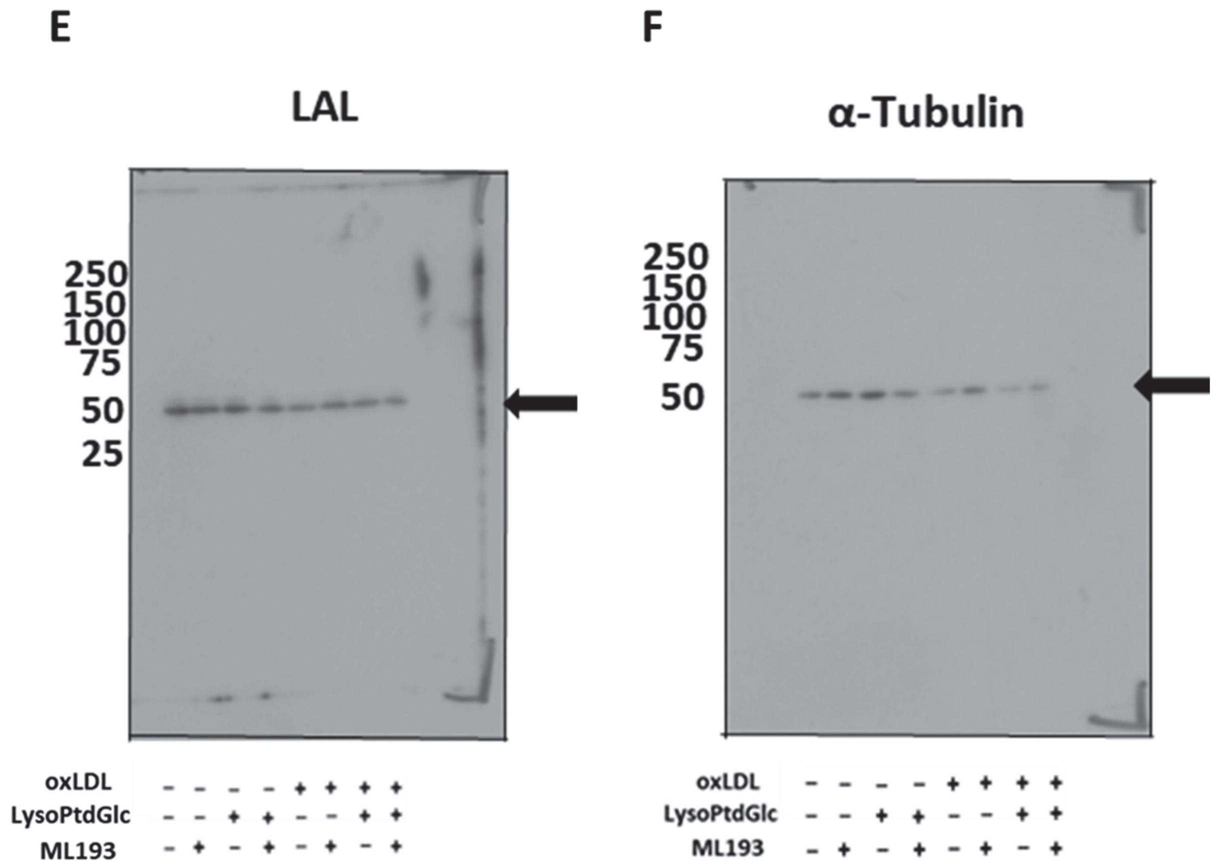

Supplement Fig 3-3. Original western blots, including protein ladders, for data presented in Fig. 4
SDS-PAGE was performed, proteins transferred onto PVDF membranes by semi-dry process, and membranes probed with appropriate HRP-conjugated primary and secondary antibodies (see M&M). Molecular weights (kDa) are indicated at left.
E, LAL F, α-tubulin.

Supplement Fig 3-4

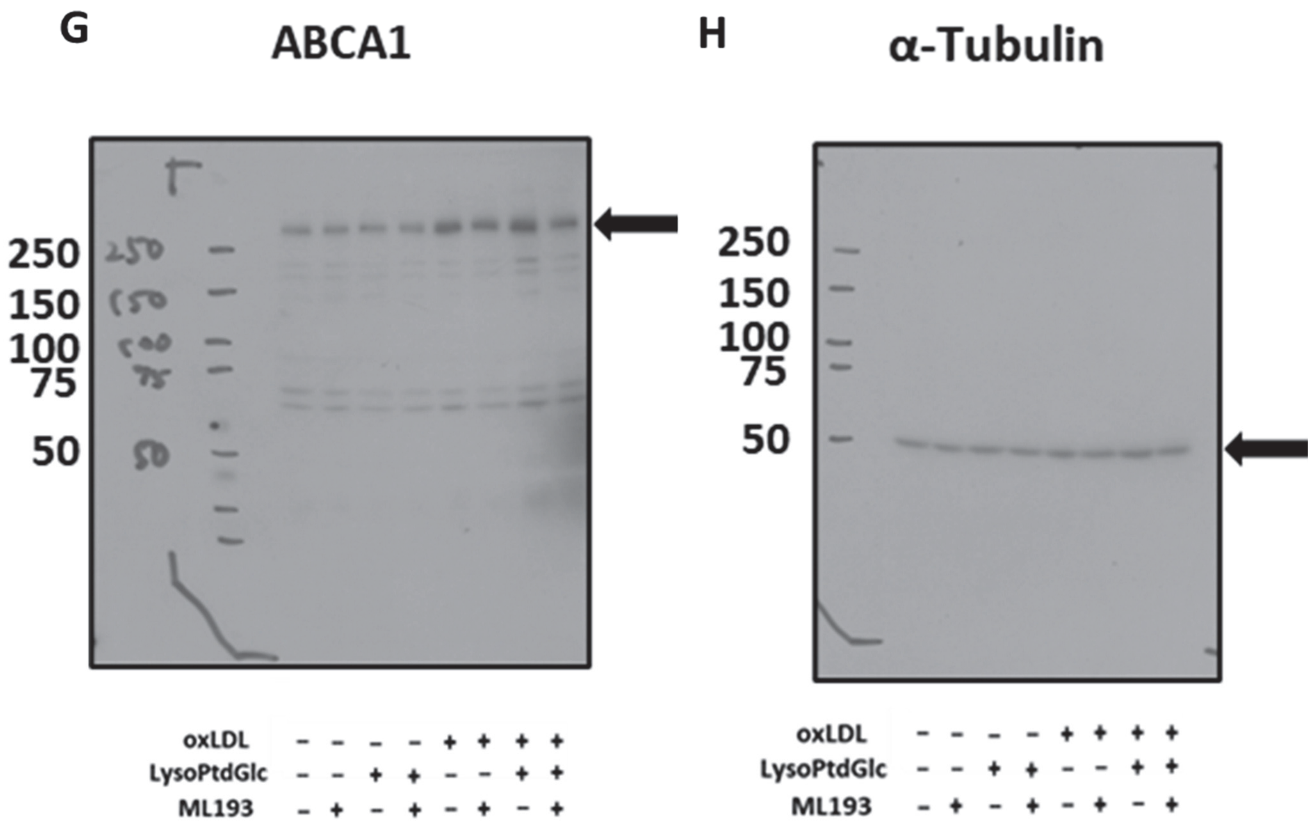

Supplement Fig 3-4. Original western blots, including protein ladders, for data presented in Fig. 4

SDS-PAGE was performed, proteins transferred onto PVDF membranes by semi-dry process, and membranes probed with appropriate HRP-conjugated primary and secondary antibodies (see M&M). Molecular weights (kDa) are indicated at left.
G, ABCA1 H, α-tubulin.

Supplement Fig 3-5

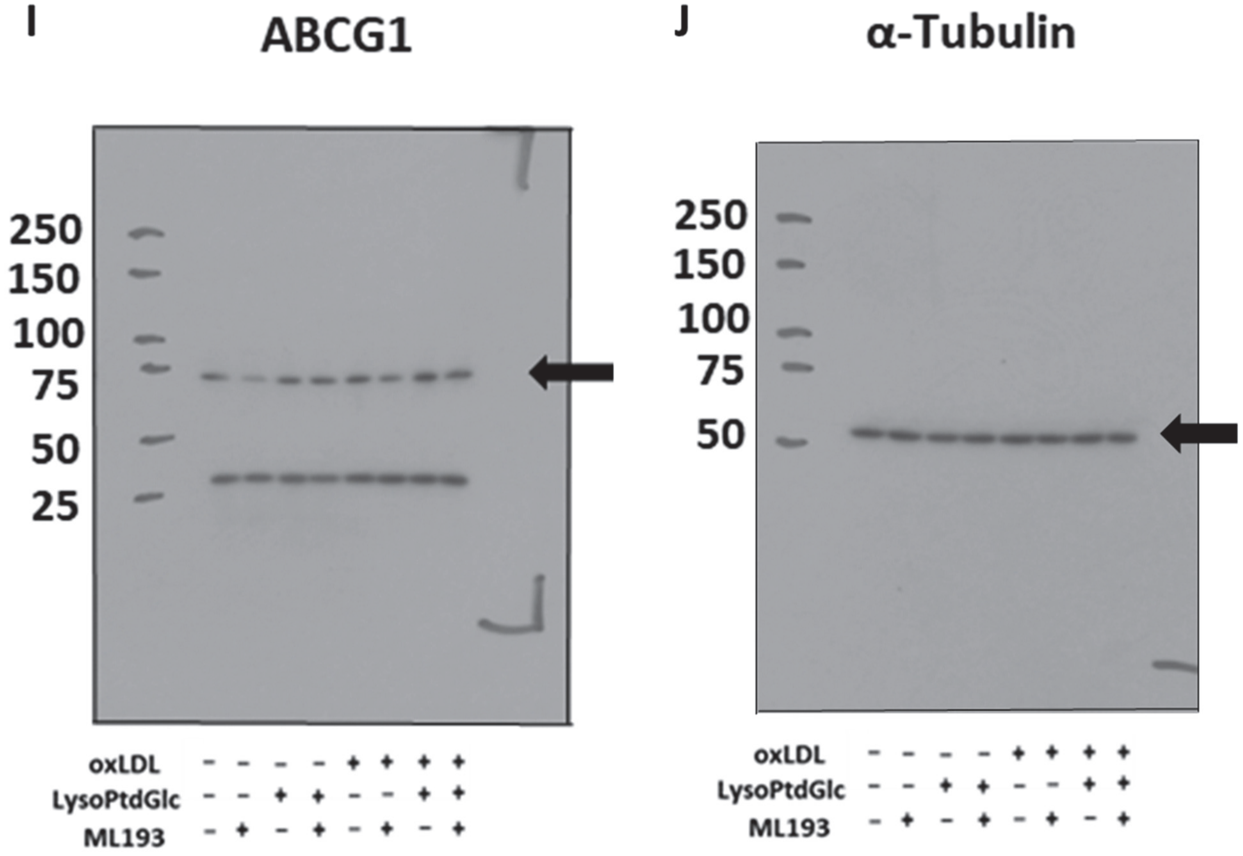

Supplement Fig 3-5. Original western blots, including protein ladders, for data presented in Fig. 4
SDS-PAGE was performed, proteins transferred onto PVDF membranes by semi-dry process, and membranes probed with appropriate HRP-conjugated primary and secondary antibodies (see M&M). Molecular weights (kDa) are indicated at left.
I, ABCG1 J, α-tubulin.

Supplement Fig 3-6

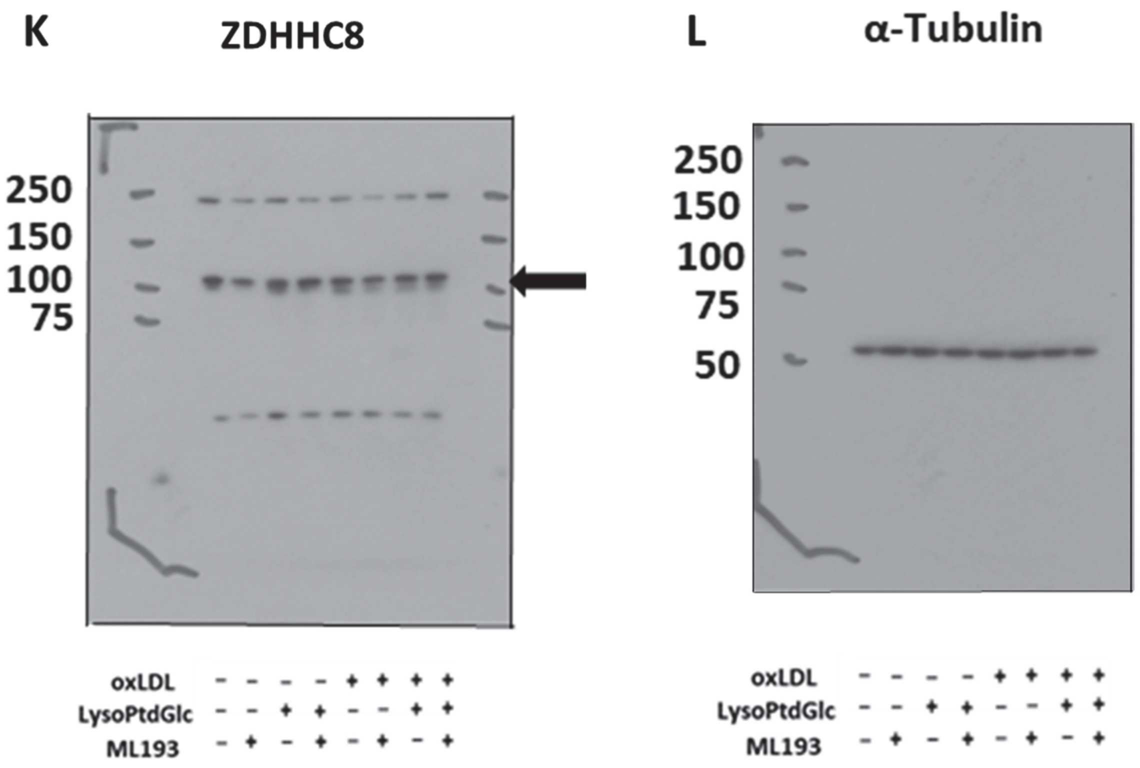

Supplement Fig 3-6. Original western blots, including protein ladders, for data presented in Fig. 4
SDS-PAGE was performed, proteins transferred onto PVDF membranes by semi-dry process, and membranes probed with appropriate HRP-conjugated primary and secondary antibodies (see M&M). Molecular weights (kDa) are indicated at left. K, ZDHHC8 L, α-tubulin.
